# Elastic Stable Intramedullary Nailing for Treatment of Pediatric Femoral Fractures; A 15-Year Single Centre Experience 

**DOI:** 10.29252/beat-070213

**Published:** 2019-04

**Authors:** Zenon Pogorelić, Tonći Vodopić, Miro Jukić, Dubravko Furlan

**Affiliations:** 1 *Department of Pediatric Surgery, University Hospital of Split, Split, Croatia*; 2 *University of Split, School of Medicine, Split, Croatia*

**Keywords:** Femur, Fracture, Children, Titanium elastic nails, Flexible intramedullary nailing, Elastic stable intramedullary nailing

## Abstract

**Objective:**

To analyze outcomes of treatment and complications in children treated with elastic stable intramedullary nailing (ESIN) due to femoral fracture.

**Methods:**

From May 2002 until May 2018 case records of 103 patients (76 male and 27 female), with median age of 9 (range, 3-17) and follow-up of 92 months, who underwent ESIN because of displaced femoral fracture were retrospectively reviewed. The patient’s information including age, sex, side involved, trauma mechanism, type of fracture, associated injuries, neurovascular status, complications, operation time, duration of hospital stay, time to implant removal were analyzed. The surgical procedure implied a reposition of bone fragments and osteosynthesis with titanium elastic nails introduced through an incision over the lateral and medial border of the distal femoral metaphysis.

**Results:**

All patients achieved complete radiographic healing at a mean of 8.5 (range, 5-15) weeks. Nine (8.49%) postoperative complications were recorded: three entry site skin irritation, two cases of valgus angulation and one case of nail protrusion, re-fracture, Varus angulation and delayed union. All complications, except case of re-fracture and one valgus angulation, were treated conservatively, with no long term consequences for the patients. Two patients were re-operated. After removal of nails all patients recovered complete function of the extremity, without long term consequences.

**Conclusion:**

The ESIN for treatment of femoral fractures shows very good functional and cosmetic results. It allows an early functional and cast-free follow-up with a quick pain reduction. Because of the excellent objective and subjective results, the operative stabilization of femoral fractures with ESIN should be recommended to all pediatrics patients

## Introduction

Fractures of the femoral diaphysis comprise around 4 % of all long-bone fractures in children [1, 2], and this is the second most frequent site affecting the lower extremity [3-5], and unlike femoral fractures in adults isolated fractures of femoral diaphysis are often seen [6]. There are several different options for treating femoral-shaft fractures in children, including early or immediate application of a hip spica cast, skeletal or skin traction followed by spica cast, minimally invasive plate osteosynthesis, elastic intramedullary nailing, external fixation, plate fixation, and internal fixation with the insertion of intramedullary nails [3, 7]. Between half and two-thirds of these fractures have a spiral configuration. These fractures should be treated by ESIN in children over three years of age, even complex spiral fractures as long as sufficient stability can be achieved. This results in rapid recovery and rehabilitation and avoids prolonged immobilisation [8]. ESIN meets all the criteria of minimally invasive bone surgery: shorter operating time, minimal soft-tissue dissection, smaller incisions and thus smaller scars, less pain, earlier mobilization and relative easy implant removal [9]. With right indications and good preoperative preparation of an experienced surgeon with this minimally invasive method of treatment is possible to achieve good bone position and stabilization appropriate to children age [9, 10]. 

We hypothesized that the ESIN as a treatment for femoral fractures in children would give satisfactory results and allow early functional follow-up with a quick pain reduction and low complication rate. The objective of this study was to evaluate the clinical and radiological outcomes and complication rates of femoral fractures treated by ESIN in children in a cohort of 103 children over the past 15 years to underline the safeness and efﬁciency of this technique. 

## Materials and Methods

 *Patients*

 The case records of 103 children (76 males and 27 females) treated for femoral fractures with ESIN, from May 2002 to May 2018 in a Department of Pediatric Surgery at University Hospital of Split, were retrospectively reviewed. The mean follow-up was 92 (3-188) months. The median age at the time of surgery was 9 (3-17) years. Total number of fractures was 106 out of 103 patients, because 3 of them had bilateral femoral fractures. Inclusion criteria were the diagnosis of femoral fracture in patients of both genders, ages 3 to 17 treated with ESIN, with minimal follow-up of 9 months. Exclusion criteria were patients older than 17 years of age, and younger than 3 year of age, patients with non-operatively treated fractures or treated operatively with method other than ESIN, patients operated in other institutions, patients with follow-up shorter than 9 months and patients with incomplete data. In all patient’s information about age, sex, side involved, trauma mechanism, type of fracture, associated injuries, neurovascular status, complications, operation time, duration of hospital stay, time to implant removal and whether the fracture was closed or open were analyzed. The study was approved by the institutional review board of our hospital. 

*Radiographic assessment*

**Fig. 1:**
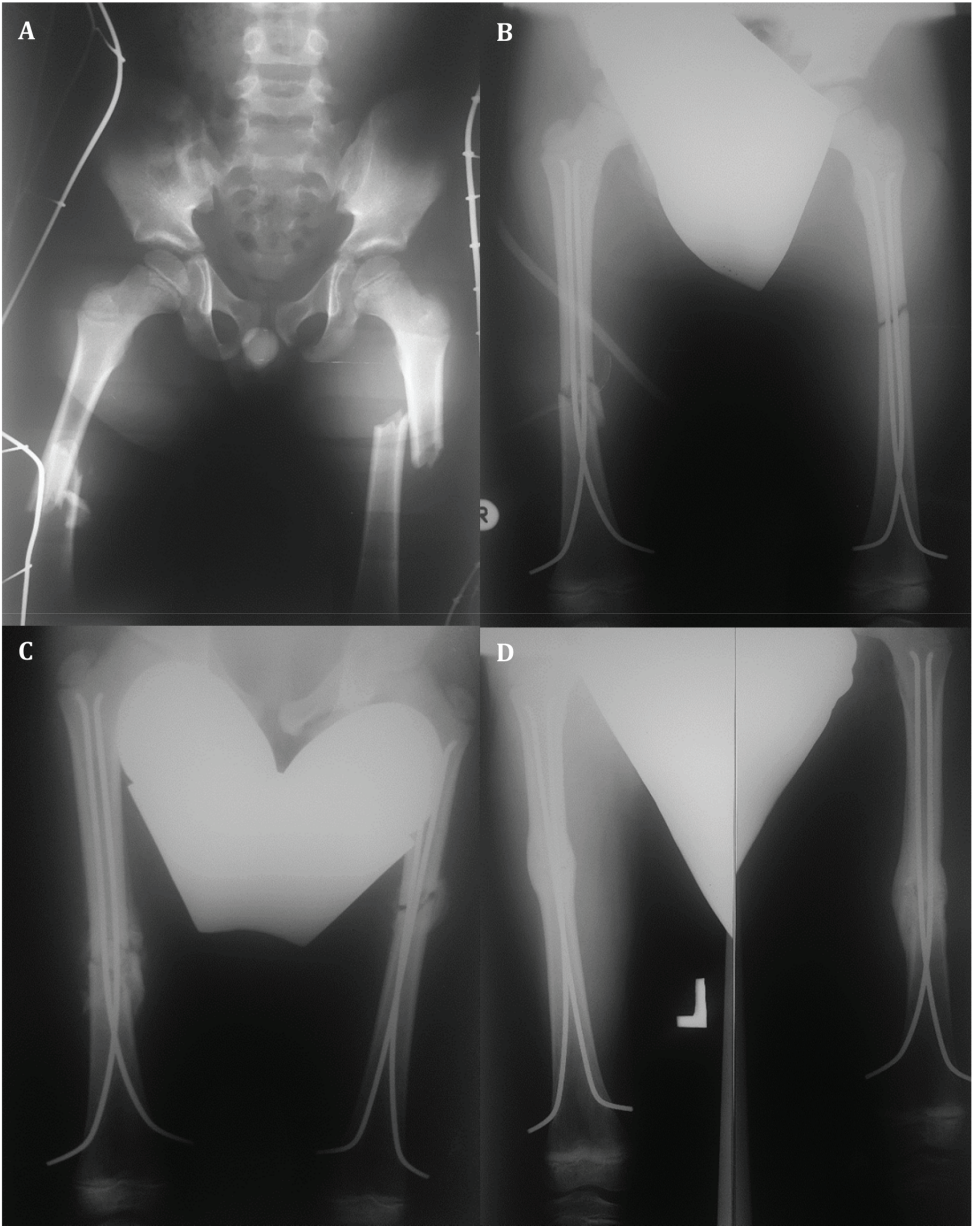
A 8-year-old boy with bilateral diaphyseal femoral fracture: **A.** Preoperative radiography; **B.** Intraoperative radiography; **C.** One month after surgery; **D.** Three months after surgery – complete healing of the fracture

 All children underwent at least full- length AP and L radiographs of the upper leg (Figure 1A). Displacement was assessed on mentioned radiographs. 

*Indications for surgery*

 All fractures of femoral diaphysis without involvement of epiphyseal growth plate. Indications for surgery were open fracture, pathological fractures and inability to achieve stable initial reduction with closed treatment.

*Surgical treatment*

 Titanium intramedullary nails (TEN Synthes® GmbH, Oberdorf, Switzerland) were used in all patients. The length of the nails was selected according to bone length and child age. Thickness of nails were determined preoperatively and intraoperatively in a way that 2 nails have to fill minimally 2/3 of medulla of most narrow part of the bone. All the children were treated under general anesthesia on a radiolucent operating table and were placed in the supine decubitus position. The fracture was first reduced by external maneuver with fluoroscopic verification. The surgical procedure implied a reposition of bone fragments and osteosynthesis with titanium elastic nails introduced through a 1.5-2 cm incision over the lateral and medial border of the distal femoral metaphysis with caution not to damage epiphyseal plate. Each fracture was treated using two flexible nails of the same diameter. The nails were shortened at the subcutaneous level. The wounds were primarily sutured. After surgery, there were no casting and physical therapy was started in early postoperative period. 

*Follow-up*

 All the patients were regularly followed clinically and radiographically 7 days, and then 1, 3, 6 months after index surgery (Figure 1B-D). Radiological evaluation was carried out using standard anteroposterior and lateral radiographs at each visit to evaluate the consolidation of the fracture and identify complications such as secondary displacement, shortening, hardware migration, delayed union, nonunion or malunion, and re-fracture. Subjective data, such as perceived pain, stiffness, and impact on daily activities, and objective data, such as range of motion, skin lesions, or surgical site infection, were recorded. Complete fracture healing was defined as a full return to activities of daily living and sports.

*Statistical analysis*

 The data were analyzed using the Microsoft Excel for Windows Version 16.0 (Microsoft Corporation, USA) and SPSS 19.0 (IBM Corp, Armonk, NY) software programs. Distributions of quantitative data were described by medians and ranges, whereas absolute rates and percentages were used to describe categorical data. Differences in median values of quantitative variables between the groups was tested with t-test. The Chi-square test with Yates correction was used for the statistical analysis of the categorical data. All values of *p*<0.05 were considered to indicate statistical significance.

## Results

Demographic and clinical data of the patients are presented in Table 1. The mechanisms of injury are presented in Table 2. The fractures in 99 children were closed, and 4 children had open fracture with no neurovascular compromise. In 89 patients the fracture was reduced by closed means, whilst in the other 14 open reductions was required due to difficulty in reduction and soft tissue interposition.

**Table 1:** Demographic and clinical data of the patients

**Fracture type**				
**Patient data**	**All fractures**	**Diaphyseal femoral fractures**	**Multifragmentary femoral fractures**	***p-value***
	(n=106)	(n=90)	(n=16)	
**Age (years)**				
Mean	9	10	9.5	0,240a
Range	2-17	2*-*17	4-17	
**Gender; n (%)**				
Male	76 (71.7)	61 (67.7)	15 (93.8)	0,095^b^
Female	27 (28.3)	26 (32.3)	1 (6.2)	
**Side; n (%)**				
Left	60 (56.6)	51 (56.6)	9 (56.3)	0,975^b^
Right	46 (43.4)	39 (43.4)	7 (43.7)	
**Operation Time (min)**				
Mean	55 ± 2.38	49 ± 3.56	60 ± 7.56	0,015a
Range	30-140	30-110	45-140	
**Hospital Stay (days)**				
Mean	6 ± 2.3	6 ± 1.4	9 ± 1.23	0,01a
Range	(4-15)	(4-13)	(7-15)	
**Complications; n (%)**	9 (8.5)	5 (5.5)	4 (25)	0,010^b^
**Time to Union (weeks)**				
Mean	8.5 ± 1.3	8 ± 1.7	10 ± 1.8	0,010a
Range	5-15	5-12	6-15	
**Follow-Up (months)**				
Mean	92 ± 12.6	93 ± 18.6	115.5 ± 20.8	
Range	3-188	3-182	8-188	
**Associated Injuries; n (%)**	42 (39.6)	31 (34.4)	11 (68.75)	0,009^b^

at-test;

b Chi square test.

**Table 2:** Distribution of fractures according to fracture type and mechanism of injury

**Fracture type**			
	All fractures	Diaphyseal femoral fractures	Multifragmentary femoral fractures
	(n=106)	(n=90)	(n=16)
**Mechanism Of Injury; n**			
Fall from standing height	33	27	6
Road traffic accident	22	20	2
Motorbike	17	12	5
Bicycle riding	10	8	2
Pathological fracture	9	9	0
Sport	8	8	0
Fall from a height	4	3	1
Fight injuries	3	3	0
**Type Of Fracture; n**			
Closed	102	86	16
Open	4	4	0

All patients achieved complete radiographic healing at a mean of 8.5 (5–15) weeks. Statistically significant difference in radiographic healing between multifragmentary and diaphyseal fractures was found (*p*=0.010). The median operative time was 49 (30-110) min for diaphyseal, and 60 (45-140) min for multifragmentary fractures (*p*=0.015). Median length of hospital stay was 6 (4-13) days for diaphyseal fracture group and 9 (7-15) days for multifragmentary group (*p*= 0.01). Complications as a result of the procedure were recorded in 9 (8.49%) patients and included: 3 entry site skin irritations, 2 valgus angulations, 1 refracture, 1 migration of the nail, 1 varus angulation, and 1 delayed healing. Patients’ characteristics, type and causes of the pathological fracture are presented in Table 3. There was no instance of loss of reduction during the post-operative period. At follow-up all patients went on to osseous union and regained a full range of movement after removal of intramedullary nails. There was no complication due to extraction of osteosynthetic material. Statistically significant difference between diaphyseal fractures and multifragmentary fractures in regards to rate of postoperative complications was found (*p*=0.010).

**Table 3:** Patients’ characteristics, type and causes of the pathological fracture

**Patient**	**Gender**	**Age**	**Side**	**Fracture type**	**Pathological cause**	**Complication**
1	F	4	R	Diaphyseal femur fracture	Osteogenesis imperfecta	/
2	F	6	R	Diaphyseal femur fracture	Osteogenesis imperfecta	/
3	F	7	R	Diaphyseal femur fracture	Osteogenesis imperfecta	/
4	M	9	L	Diaphyseal femur fracture	Cyst	/
5	F	12	R	Diaphyseal femur fracture	Osteogenesis imperfecta	/
6	M	12	R	Diaphyseal femur fracture	Cyst	delayed union
7	F	13	R	Diaphyseal femur fracture	Osteogenesis imperfecta	/
8	M	13	L	Diaphyseal femur fracture	Cyst	refracture
9	M	17	L	Diaphyseal femur fracture	Cyst	/

Totally, two children required reoperation, one with refracture and one with valgus angulation. Other complications were treated conservatively. A 13-year-old boy with fracture of the femur due to simple bone cyst developed refracture of three weeks after removal of the nails. Before nails removal he had satisfactory radiological healing and he fell down three weeks after nails removal. Closed reduction and osteosynthesis with titanium nails was peformed. At follow-up he had uneventful course. Another reoperated child was 14-year-old girl with valgus angulation of the distal femur following titanium intramedullary nailing for metaphyseal-diaphyseal fracture. At follow-up she developed non-tolerante distal femoral valgus deformity. Osteotomy and plate fixation was performed. At follow-up she had uneventful course. The implants were removed under general anesthesia as a day surgery procedure without difficulty at a median time of seven (6-12) months from the index surgery. 

## Discussion

Long bone fractures are among the most common emergencies in most trauma centers of the world. Although, in children, such fractures can usually be successfully treated with proper closed reduction and immobilization, there are cases where surgical stabilization and osteosynthesis is needed. Historically, long bone fractures in children were only treated immobilized immediately after injury or after a certain time of traction. Surgical treatment was limited to open fracture or politraumatised patients with fractures of long bones. Since recently, the number of surgical procedures has increased as well the indications that also include isolated fracture of femur bone. The ESIN has been promoted to the real gold standard of long bone fractures treatment in children. This method has gained wide popularity for clinical efficacy and low risk of complications in long bone fractures treatment. Many studies support the use of this method in femoral fractures, emphasizing the advantages of closed reduction and avoiding damage to epiphyseal growth plate when laying the nails [11, 12]. However, when using the ESIN, there is a possibility of complications, and frequency of ones varies according to the experience of the operator surgeon, the proper technique of performing and settings of incorrect indication for this method. The most commonly reported complications include infection, granuloma formation, skin irritation at the nail entry site and refracture [12-14]. As previously mentioned, femoral diaphysis fractures account for around 4% of all long-bone fractures in children [1, 2] and are on third place of diaphyseal fractures in children only after forearm and tibial diaphyseal fractures [6].

Surgical fixation with early mobilization has been recognized as a superior mode of treatment over the past two decades and its usage is increasing. Symanovsky *et al*. reported 13 cases of femoral diaphyseal fractures treated with closed reduction and ESIN in children 3 to 5 years of age. As reported, none of their subjects had poor or reduced healing or osseous nonunion. Only two minor complications were recorded. Although it is not recommended to perform surgery in children of this age with simple femoral fracture, it may be a good treatment in children where immobilization did not achieved treatment success [15]. Buechsenschuetz et al. compared the treatment for femoral fracture by traction and immobilization in relation to the ESIN. They concluded that the cost of treatment with the ESIN was lower and the patients were better able to bear the appearance of the scarring, and the parents were ultimately more satisfied [16]. 

Khazzam *et al*. described the use of titanium elastic nails for the femoral fracture in 135 patients. There were a total of 138 fractures with a average follow up of 15.6 months and an average age of patients was 9.7 years. They reported 16 complications (11.5%), of which 3 refractures, 2 cases of delayed healing, 3 varus or valgus angulation, 5 cases of skin irritation at the nail entrance site and 1 asymptomatic proximal miration the nail. These results show the success of using the ESIN in femoral fractures, regardless of the age, type and location of the fracture [17]. 

Rapp *et al*. reported the association of increased body mass with the frequency of complications and final outcome. They concluded that children older than 10 years with a body mass greater than 50 kg and with still opened epiphyseal growth plates are more susceptible to complications, regardless of the treatment technique choice. However, a lower rate of similar complications was observed in patients treated with the ESIN [18].

The major problem of pediatric traumatology is the multifragmentary diaphyseal long bone fractures. These fractures are associated with a high percentage of complications and a poor final outcome. Aksoy *et al*. reported the treatment of 9 complicated cases of femoral fracture by the ESIN, previously treated with an external fixator. The average age of patients was 8.7 years, and the average follow-up was 46.6 months. Full healing was achieved after 6-9 months. No infections nor neurovascular injuries have been reported in the study which indicate that ESIN is a good choice the treatment of complicated femoral fractures [19].

Simple bone cysts are cavities filled with liquid, typically located in the metaphysis of long bones in children and adolescents. The proximal humerus and femur are the most commonly involved regions, followed by calcaneal and ilium bone [20]. Common complications of such cysts are pathological fractures. Pogorelić *et al*. reported that the ESIN has dual advantages, firstly continuous decompression and drainage of the cyst, and early direct bone segment stability, enabling early mobilization and the return to normal activities in prepubertal patients [21]. In our study a total of 4 patients were treated with ESIN for the pathological fracture of the femur based on underlying juvenile cyst. All patients had complete bone healing and regression of the cyst. 

In our study, 103 children were operated by the ESIN due to a total of 106 femoral fractures. The average age at the operation was 9 years. A closed reduction was performed in 89 patients, while in the other 14, an open reduction was required due to the difficulty in repositioning and soft tissue interposition. Total of 9 postoperative complications (8.49%) were recorded. No intraoperative complications were recorded. Most common complication was entry site skin irritation in 3 cases, 2 cases of valgus angulation and one case of nail protrusion, refracture, varus angulation and delayed union/ healing. At follow-up all patients went onto complete osseous union which was radiologically confirmed in average time of 8.5 weeks and regained full range of movements after removal of intramedullary nails. Recorded rate of complications was lower in our study compared to other mentioned studies, probably because of good and proper indication for surgery and large experience od surgeons.

Based on results of our study we can conclude that ESIN satisfies all criteria of minimally invasive bone surgery, and it is shown that, for diaphyseal femoral fractures in children, it is very efficient method with excellent functional and esthetic outcomes and low rate of complications if indications and biomechanical principles are respected.

## Conflict of Interest:

None declared.
